# Resilience among parents whose child died of cancer – investigating its role on psychological distress and prolonged grief disorder: results from a cross-sectional survey in Switzerland

**DOI:** 10.1186/s12904-025-01854-8

**Published:** 2025-08-04

**Authors:** Peter Francis Raguindin, Eva De Clercq, Anna Katharina Vokinger, Eddy Carolina Pedraza, Céline Bolliger, Katrin Scheinemann, Eva Maria Tinner, Eva Bergstraesser, Andre Oscar von Bueren, Gisela Michel

**Affiliations:** 1 Faculty of Health Sciences and Medicine, University of Lucerne, Alpenquai 4, Lucerne, 6005 Switzerland; 2 Division of Hematology-Oncology, Children’s Hospital of Eastern Switzerland, Claudiusstrasse 6, St Gallen, 9006 Switzerland; 3 Division of Hematology/Oncology, Department of Pediatrics, Inselspital Bern, University of Bern, Freiburgstrasse 15, Bern, 3010 Switzerland; 4 University Children’s Hospital Zurich, Zurich, Switzerland; 5 CANSEARCH Research Platform for Pediatric Oncology and Hematology, Faculty of Medicine, Department of Pediatrics, Gynecology and Obstetrics, University of Geneva, Rue Willy-Donzé 6, Geneva, 1211 Switzerland

**Keywords:** Bereavement, Parents, Resilience, Childhood cancer

## Abstract

**Introduction.:**

The death of a child profoundly impacts parents, often leading to anxiety, depression, and posttraumatic stress. However, factors such as resilience—defined as the capacity to adapt to adversity—are less explored. Resilience may mitigate grief-related distress. This study aimed to: (a) describe and compare resilience levels between bereaved parents and parents of childhood cancer survivors (CCS parents), (b) identify sociodemographic and cancer-related determinants of resilience among bereaved parents, and (c) investigate resilience’s association with psychological distress and prolonged grief disorder.

**Methods.:**

This is a cross-sectional study done in Switzerland. Bereaved parents were recruited from three hospitals and through patient advocacy groups. CCS parents were taken from a nationwide, population-based study (Swiss Childhood Cancer Survivor Study-Parents). Resilience, psychological distress, and prolonged grief disorder were assessed using the Connor-Davidson Resilience Scale (CD-RISC 10), Brief Symptom Inventory-18 (BSI-18), and Prolonged Grief 13 (PG-13), respectively. Regression analysis (linear and logistic) was used to identify factors associated with resilience, and the association of resilience with psychological distress and prolonged grief disorder (adjusted for age, sex, and time since death).

**Results.:**

We included 103 bereaved parents and 345 CCS parents in the analysis. Bereaved parents were younger compared to CCS parents (mean 53.7, SD 8.3 vs. 62.5, SD 6.7 years, *p* < 0.001), with both groups predominantly female (66% and 58%). Bereaved parents reported higher resilience scores than CCS parents (crude mean: 29.8 vs. 27.9; *p* = 0.005), with adjusted analyses confirming this finding. High risk of poverty was the only significant determinant of lower resilience (adjusted β = -7.37, 95% CI: -11.21, -3.54; *p* < 0.001). Higher resilience was associated with lower likelihood to report psychological distress (adjusted OR = 0.83, 95% CI: 0.74, 0.93, *p* = 0.002) and prolonged grief disorder (adjusted OR = 0.73, 95% CI: 0.58, 0.92, *p* = 0.008).

**Conclusion.:**

Bereaved parents exhibited higher resilience than CCS parents. Resilience in bereaved parents was strongly associated with reduced psychological distress and lower likelihood of having prolonged grief disorder. Targeting modifiable factors such as poverty may enhance resilience and thereby help to improve mental health outcomes for bereaved parents.

**Supplementary Information:**

The online version contains supplementary material available at 10.1186/s12904-025-01854-8.

## Introduction

The death of a child from cancer is a devastating experience for parents [1, 2]. Around 10–20% of parents suffer from emotional turmoil resulting in adverse psychological outcomes, such as anxiety, depression, and post-traumatic stress [3, 4]. And while grief is a normal reaction to loss, prolonged grief is seen in 10% of the general population [5] and occurs in substantially higher rates in bereaved parents [6]. Other long-term effects child loss on parents include marital and family problems, socio-economic hardships [7], and an overall lower quality of life [8]. However, few studies have investigated the impact of the child’s death on parental resilience [1, 2, 9].

The American Psychological Association defines resilience as “the process of adapting well in the face of adversity, trauma, tragedy, threats or significant sources of stress” [10]. It is an internal resource that could protect a person from developing psychological distress, allowing them to cope successfully when faced with subsequent stresses in life [1, 2, 11]. Previous studies have shown that parents may develop resilience after the death of their child [2, 9, 12]. Social support and strong partner relationships were identified as factors associated with resilience among bereaved parents [2, 12, 13]. Studies have further shown that bereaved parents who present high levels of resilience are less likely to suffer from depression and anxiety and have higher social functioning [12, 14].

There are various gaps in the existing literature on resilience and bereaved parents. First, the impact of child loss on parental resilience has rarely been explored quantitatively [1, 2, 9]. Quantitative analysis is needed so that sociodemographic and cancer-related factors influencing resilience can be investigated more systematically, and comparison of relevant population can be done. Second, it is still unclear whether there are differences in parental resilience according to the survival outcome of their child with cancer (mortality versus survival). Most studies had no comparison group or had chosen parents of healthy children as comparison [2, 13]. Knowing the difference in resilience on special groups would help tailor support strategies. Third, few studies have investigated the association of parental resilience and psychosocial outcomes after their child’s death from cancer [12, 15]. Finally, most studies have focused on short-term bereavement (< 3 years after death) [12, 16] and thus did not investigate the long-term effects of child’s death up to 10 years or more [2, 9].

We aimed to (a) describe the level of resilience of bereaved parents and compare it with that of parents of childhood cancer survivors, (b) identify sociodemographic and cancer-related factors potentially influencing resilience among bereaved parents and examine their association with time since the child’s death, and (c) investigate the association of resilience with psychological distress and prolonged grief disorder among bereaved parents.

## Methods

We conducted a cross-sectional survey among bereaved parents and parents of childhood cancer survivors (CCS parents). We have presented our study following the Strengthening the Reporting of Observational Studies in Epidemiology (STROBE) guidelines [17] (See Appendix).

### Sample and procedure

For the bereaved parents, eligible participants included parents of children who (a) were diagnosed with cancer, as defined by the International Classification of Childhood Cancer, Third Edition (ICCC-3) [18], (b) were ≤ 18 years of age at time of diagnosis, (c) received treatment in Switzerland, and (d) died of cancer at least one year prior to study participation. To identify potential participants, the Swiss Childhood Cancer Registry (ChCR) provided a list of eligible cases to the participating hospitals from German-speaking regions that were involved in the study. These hospitals confirmed the parents’ contact information through their records, excluding individuals deemed unsuitable for participation due to personal or medical reasons, such as vulnerability or distress, and sent eligible parents the study invitation. The invitation package included study information and written consent, questionnaires (one per parent), and prepaid return envelopes. Parents were encouraged to complete the questionnaires individually, ensuring independent responses from both parents. They were given the option to complete either a paper or an online questionnaire (Qualtrics™, Provo, Utah, US). The survey was conducted from July 2022 to July 2023. The hospitals followed up with non-responders by making reminder phone calls starting earliest five weeks after the initial mailing. Additionally, cancer and bereavement support organizations helped disseminate information about the study.

Responses from printed questionnaires were manually entered into the database by research assistants, with an accuracy check performed on 14.5% of the entries (*N* = 15). Double entry by two independent study staff showed high accuracy (kappa = 0.93).

For CCS parents, we used data from a previously conducted nationwide population-based study, the Swiss Childhood Cancer Survivor Study – Parents (SCCSS-Parents) [19]. Parents in this study were eligible if their child: (a) was diagnosed with cancer according to ICCC-3 between 1976 and 2009, (b) aged ≤ 16 years (according to inclusion criteria of the Swiss Childhood Cancer Registry), and (c) Swiss resident at diagnosis, (d) had survived at least five years post-diagnosis, and (e) was aged ≥ 20 years at the time of study participation (following the minimum age requirement for study participation of the survivors). Eligible survivors were identified through the Childhood Cancer Registry (ChCR), and addresses were verified via an online telephone directory. Parents were invited to participate through their former treating hospital, which sent study information two weeks before mailing questionnaires. Non-responders received reminders after four to six weeks and again after an additional eight weeks. The SCCSS-Parents survey was conducted from January 2017 to February 2018. For this analysis, we used the data from CCS parents from German-speaking regions and age-range matched with bereaved parents.

### Measures

Resilience was assessed using the Connor-Davidson Resilience Scale (CD-RISC 10), a validated self-report tool measuring an individual’s “ability to adapt to adversity” [11, 20]. This is one of the most commonly used scales to measure resilience in survivors and their families [21]. This scale consists of 10 items rated on a 5-point Likert scale (0 = not true at all to 4 = true nearly all the time), yielding a sum score ranging from 0 to 40. Scores were computed by summing the responses, with missing responses (≤ 2 items) imputed using the mean of the remaining items. If ≥ 8 responses were missing, the score was considered invalid. We included three additional questions from long form of CD-RISC (25 items) [11]. These questions were related to spirituality and religiosity.

Psychological distress was measured using the Brief Symptom Inventory (BSI-18) [22, 23], a psychometric tool validated in the Swiss population [24]. This BSI-18 assesses depression, anxiety, somatization, and overall psychological distress (Global Severity Index, GSI) using 18 items scored on a 5-point Likert scale (0 = not at all to 4 = extremely). Total scores were converted into T-scores (mean = 50, SD = 10) based on norms established in the German population [23]. Caseness indicating significant psychological distress was defined as a GSI T-score ≥ 63 or a T-score ≥ 63 on at least two of the three subscales (depression, anxiety, somatization). Missing subscale responses (1 item) were imputed using the subscale mean, while missing values exceeding this threshold rendered the subscale invalid for inclusion in the analysis [22, 23].

Prolonged grief disorder is a condition of intense grief and disrupting daily life persisting for more than one year after loss for adults, or six months for adolescents and children. This was assessed using Prolonged Grief 13 (PG-13) [25], included five main components: (a) bereavement event, (b) separation distress, (c) duration of grief, (d) cognitive/emotional/behavioral symptoms, and (e) functional impairment [26]. Bereaved parents automatically fulfilled the bereavement event criteria due to the study’s inclusion criteria. Separation distress was evaluated using two items on yearning and emotional pain, with responses rated on a 5-point Likert scale (1-not at all, to 5-several times a day). The duration of grief is defined by the occurrence of daily feelings of separation distress persisting for at least six months after the loss (yes/no). Cognitive, emotional, and behavioral symptoms were measured using nine items, using 5-point Likert scale (1-not at all, to 5- overwhelmingly). Impairment in social, occupational, or other areas of functioning was assessed using a binary yes/no response. A classification of prolonged grief disorder required meeting all criteria. We did not impute missing responses.

Sociodemographic variables collected were sex (male, female), age (< 50 years, 50–60 years, > 60 years old), education (compulsory education, vocational training, upper secondary/university), employment (unemployed which includes invalidity claim, housemaker or retired, versus employed which included full-time or part-time employment), risk of poverty (for those living as parent-couples CHF < 6000, for single-parents CHF < 4500; this cut-off was chosen based on the published risk-of-poverty of the Swiss Federal Statistical Office [27] which was also used in a previous publication [28]), living arrangement (asked if living alone, versus ≥ 2 persons in the household), having a religion (yes, no), partnership status (yes [in a partnership], no), and migration background (defined as not being a Swiss citizen, not a Swiss citizen since birth, or a Swiss citizen not born in Switzerland). Child- and cancer-related information, i.e. the child’s sex (male, female), age at diagnosis (< 5 years, 5–9 years, ≥ 10 years), cancer type (leukemia/lymphoma, central nervous system tumor, and other solid tumors), and time since diagnosis (< 20 years, ≥ 20 years). For bereaved parents, bereavement-specific variables, including the child’s age at death (< 5 years, 5–9 years, ≥ 10 years), location of death (home versus hospital or health facility), and time since death (< 10 years, ≥ 10 years), were also reported by the parents in the questionnaire.

### Data analysis

We described the sociodemographic and cancer-related characteristics of both bereaved parents and CCS parents using means and SD or proportions. Bereavement or death related characteristics were only described for bereaved parents.

We followed the analysis framework in Appendix Figure S1. To compare resilience levels (resilience sum score and individual items) among bereaved and CCS parents (Aim 1), we used Student’s t- test for continuous variables and chi-square tests for categorical responses. Marginal means were also computed using linear regression, adjusting for sociodemographic variables that were statistically different between two groups (decided a posteriori from bivariate comparison analysis with p value < 0.05).

To identify determinants of resilience (Aim 2), we used univariable linear regression using resilience as dependent variable (outcome) and the sociodemographic, child- and cancer-related characteristics as independent variables (predictors). Variables with *p* < 0.10 were included in multivariable linear regression models to identify independent predictors. We also explored non-linear associations between time since death and resilience sum score using restricted cubic spline regression for bereaved parents. We plotted a spline modeling using resilience sum score as dependent variable (outcome) and time since death as independent variable (predictor). Penalization and smoothing parameters were applied, and graphical plots were generated to visualize these relationships. Missing data were handled using full information maximum likelihood, assuming data were missing at random. Additional questions on religiosity and spirituality were also described.

Finally, to determine the association between resilience (Aim 3), and psychological distress and prolonged grief disorder, regressions were also fitted using linear regression for continuous outcomes (GSI-18, anxiety, depression, and somatization) and logistic regression for binary outcomes (Caseness with psychological distress, prolonged grief disorder). Separate models were fitted for each subscale of psychological distress, with crude and adjusted estimates reported. Age, sex, and time since death were included in adjusted models to minimize bias.

As a sensitivity analysis, multilevel linear regression models with random intercepts were fitted to account for within-household (within partnership) clustering. Resilience sum score was used as the dependent variable. Independent variables included the study population (Aim 1) and sociodemographic, child-, and cancer-related characteristics (Aim 2). The childhood cancer patient identification was modeled as a second level cluster to account for non-independence of observations within household (within partners).

All analyses were conducted using Stata 18.5 (StataCorp, TX). Statistical significance was set at *p* < 0.05 for two-tailed tests. All analyses are exploratory, and no adjustments for multiple testing were applied.

### Ethical considerations

Ethical approval for the Bereaved parents survey was granted by the Ethics Committee of Northwest and Central Switzerland (EKNZ 2021 − 00906, 4 August 2021). Similarly, ethical approval for the SCCSS-Parents study was obtained from the same committee (EKNZ 2015-075, 26 March 2015).

## Results

**Fig. 1:**
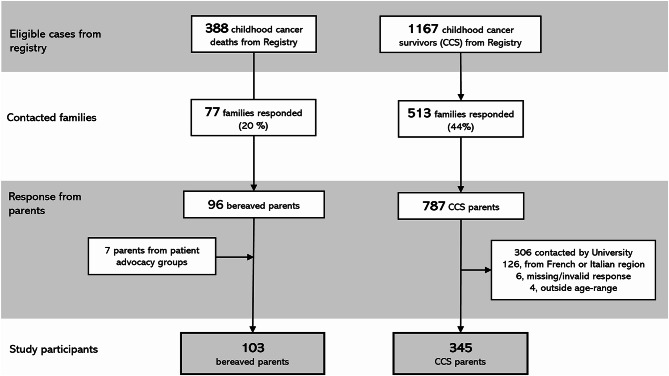
Flowchart of study participants

The study population included 103 bereaved parents (of 81 childhood cancer deaths) and 345 parents (of 217 cancer survivors) (Table 1; Fig. 1**)**. Participating bereaved parents were younger on average (mean age 53.7 years, SD 8.3) compared to CCS parents (mean age 62.5 years, SD 6.7, *p* < 0.001). Both groups were predominantly female, with similar levels of vocational and higher education. Employment rates were comparable (25.5% vs. 26.0%), but a lower proportion of bereaved parents were at risk for poverty compared to CCS parents (11.8% vs. 31.3%, *p* < 0.001). Religion and migration background differed significantly, with fewer bereaved parents having a religion (69.6% vs. 82.3%, *p* = 0.005) and more bereaved parents with migration background (20.0% vs. 8.2%, *p* = 0.003) (Table 1).

**Table 1:** Characteristics of study participants

	Bereaved parents *N* = 103	Parents of childhood cancer survivors (CCS parents) *N* = 345	*p*-value^1^
Sociodemographic characteristics			
Sex			
• Male	35 (34.0%)	145 (42.0%)	0.144
• Female	68 (66.0%)	200 (58.0%)	
Age in years (mean, SD)^2^	53.7 (8.3)	62.5 (6.7)	< 0.001
Age categories			
• < 50 years	30 (29.7%)	4 (1.2%)	< 0.001
• 50–60 years	45 (44.6%)	128 (37.3%)	
• > 60 years	26 (25.7%)	211 (61.5%)	
Education			
• Compulsory education	5 (4.9%)	32 (10.0%)	0.188
• Vocational training	66 (64.7%)	181 (56.7%)	
• Upper secondary or university education	31 (30.4%)	106 (33.2%)	
Employment status^3^			
• Employed	76 (74.5%)	248 (74.0%)	0.923
• Unemployed	26 (25.5%)	87 (26.0%)	
Risk of poverty^4^			
• Low risk	90 (88.2%)	224 (68.7%)	< 0.001
• High risk	12 (11.8%)	102 (31.3%)	
Living arrangement			
• Alone	5 (4.9%)	25 (7.7%)	0.339
• Shared	97 (95.1%)	301 (92.3%)	
Having a religion^5^			
• With religion	71 (69.6%)	284 (82.3%)	0.005
• Without religion	31 (30.4%)	61 (17.7%)	
Partnership status			
• No	8 (7.8%)	30 (9.0%)	0.709
• Yes	94 (92.2%)	302 (91.0%)	
Migration background^6^			
• No	60 (80.0%)	301 (91.8%)	0.003
• Yes	15 (20.0%)	27 (8.2%)	
Clinical and cancer-related characteristics			
	*N* = 81 cancer deaths	*N* = 217 cancer survivors	
Child’s sex			
• Male	46 (56.8%)	116 (53.5%)	0.607
• Female	35 (43.2%)	101 (46.5%)	
Child age at diagnosis			
• < 5 years	31 (38.3%)	85 (39.2%)	0.986
• 5–9 years	22 (27.2%)	59 (27.2%)	
• ≥ 10 years	28 (34.6%)	73 (33.6%)	
Cancer diagnosis			
• Leukemia/lymphoma	22 (27.2%)	118 (54.4%)	< 0.001
• CNS tumor	36 (44.4%)	24 (11.1%)	
• Other solid tumors	23 (28.4%)	75 (34.6%)	
Time since diagnosis, years (mean, SD)^2^	20.4 (7.6)	23.4 (7.3)	0.001
Time since diagnosis			
• < 20 years	38 (46.9%)	63 (29.0%)	0.004
• >/= 20 years	43 (53.1%)	154 (71.0%)	
Bereavement or death-related characteristics			
Age at death			
• < 5 years	23 (22.6%)		
• 5–9 years	28 (27.5%)		
• ≥ 10 years	51 (50.0%)		
Location of death			
• Home	45 (55.6%)		
• Healthcare facility	36 (44.4%)		
Time since death, years (mean, SD)	11.0 (5.5)		
Time since death			
• < 10 years	35 (43.8%)		
• ≥ 10 years	45 (56.2%)		

Abbreviations: CCS, childhood cancer survivor; CNS, central nervous system; SD, standard deviation

^1^ *p*-values were derived from chi-squared test for comparison of proportion, unless specified otherwise

^2^ *p*-values for continuous variables were obtained from Student’s t-test

^3^ Employment status: Employed included those with full-time or part-time employment. Unemployed included those who are on unemployed, on invalidity claims, in training/education, or retired

^4^ Risk of poverty: For those parent-couples CHF < 6000/month, for those single-parents CHF < 4500/month. This were chosen based from the published risk-of-poverty of the Swiss Federal Office of Statistics which are CHF 3,933/month for single parents with two children aged < 14 years and CHF 5,163/month for parent couples and was also used in a previous publication

^5^ Having a religion: Participants who indicated a religious denomination were classified as “with religion”. Those who identify as with no religion, or had no answer were classified as “without religion”

^6^ Migration background: We classified individuals with migration backgrounds as those who are not Swiss citizens, were not born in Switzerland, or were not Swiss at birth

A diagnosis of CNS tumors (44.4%) was higher in children of bereaved parents compared to children of CCS parents, where diagnoses of leukemia/lymphoma dominated (54.4%, *p* < 0.001) (Table 1). The average time since diagnosis to survey date was shorter for bereaved parents (20.4 years, SD 7.6) than for CCS parents (23.4 years, SD 7.3, *p* < 0.001). For bereaved parents, the average time since their child’s death was 11.0 years (SD 5.5). More deaths occurred at home (55.6%) than in healthcare settings. Additionally, 50% of children died after the age of 10 years.

### Comparison of resilience (Aim 1)

**Fig. 2:**
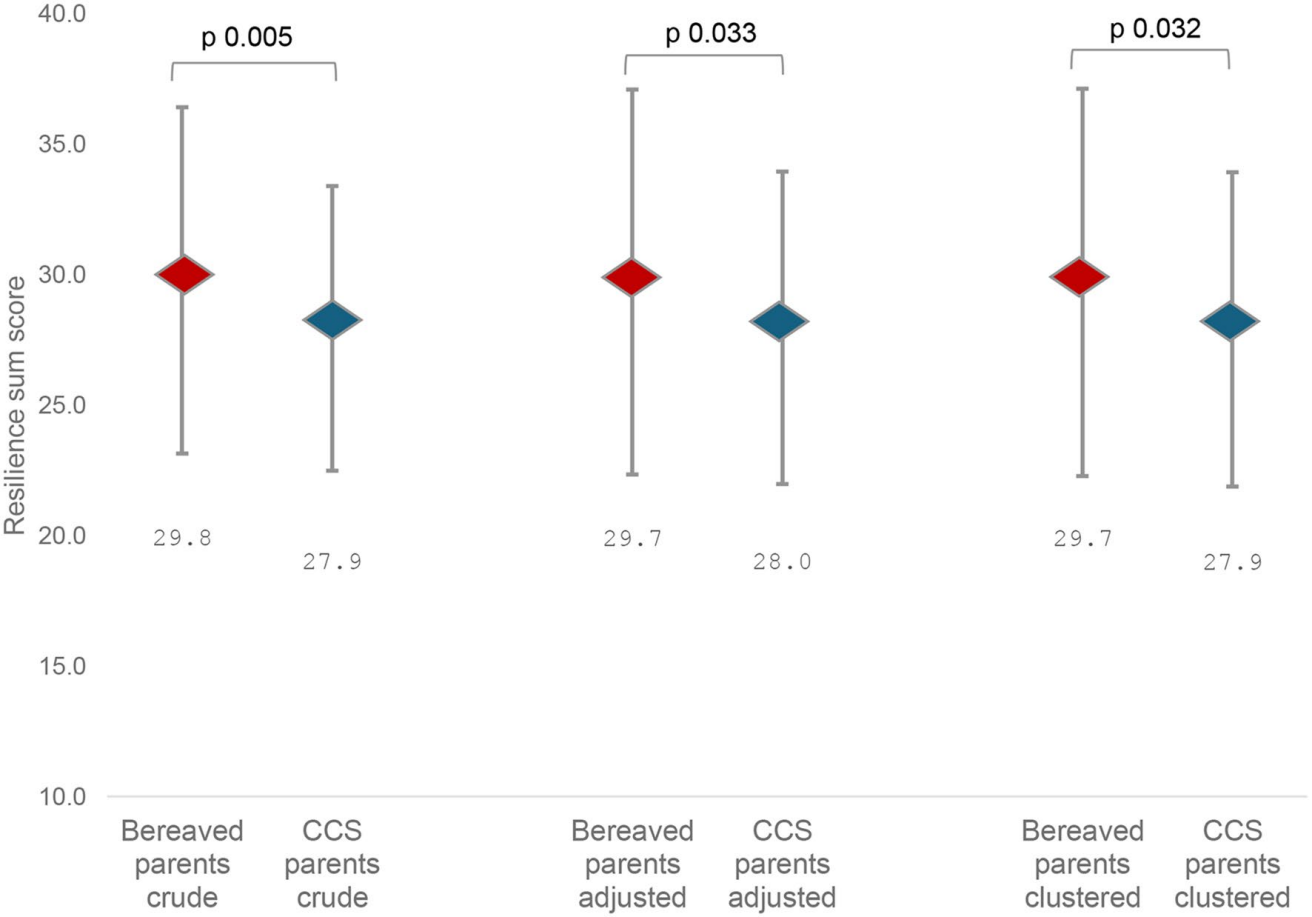
Comparison of resilience between bereaved parents and CCS parents (covariate adjustment in a linear regression for factors that are different between groups, namely, age, risk of poverty, religion, migration, time since diagnosis, clustered analysis according to household/partnership)

The resilience sum score was significantly higher among bereaved parents compared to CCS parents (mean 29.8, SD 6.6 vs. mean 27.9, SD 5.5, *p* = 0.005) (Fig. 2). Item comparison can be found in Appendix Table S1. Adjusted resilience sum scores (adjusted for different ages, risk of poverty, having a religion, and migration background distribution), consistently revealed higher resilience in bereaved parents compared to CCS parents (Fig. 2). Findings from the multilevel model showed similar findings (Appendix Table S2).

Comparisons of additional resilience items on religiosity and spirituality are described in Appendix Table S3. Bereaved parents exhibit a bimodal distribution in their responses to the religiosity question. They have a higher proportion of individuals who do not believe in God (42.7% vs. 29.9%) and a higher proportion of believers (20.4% vs. 13.9%) compared to CCS parents. Bereaved parents reported lower levels of a strong sense of purpose in life compared to CCS parents (*p* < 0.001), while they were more likely to report having at least one close and secure relationship (*p* = 0.017). There is no difference between belief in fate or God which helps them in their life (*p* = 0.374).

### Determinants of resilience in bereaved parents (Aim 2)

Among sociodemographic variables, only risk of poverty was significantly associated with resilience (Table 2). Parents at high risk of poverty had a lower resilience sum score compared to those at low risk for poverty (Univariable regression coefficient: -7.86, 95% CI: -11.59 to -4.13, *p* < 0.001 multivariable model coefficient: -7.37, 95% CI: -11.21 to -3.54, *p* < 0.001) (Table 2). None of the cancer-related characteristics showed significant associations with resilience (Table 2). Findings from the multilevel model showed similar findings (Appendix Table S4).

**Table 2:** Determinants of resilience for bereaved parents

			Univariable regression^1^			Multivariable regression^2^			
	Mean score^3^		Coefficient	95% Confidence interval	*p*-value	Coefficient	95% Confidence interval	*p*-value	
*Sociodemographic characteristics of bereaved parents*									
Sex (male)	30.8								
• Female	29.2		-1.55	(-4.28, 1.18)	0.263				
Age (< 50 years)	28.4								
• 50–60 years	30.2		1.83	(-1.26, 4.92)	0.242				
• > 60 years	30.6		2.21	(-1.30, 5.72)	0.215				
Risk of poverty (low risk)	30.6								
• High-risk	22.7		**-7.86**	**(-11.59**,**-4.13)**	**< 0.001**	**-7.37**	**(-11.21**,** -3.54)**	**< 0.001**	
Living arrangement	27.2								
• Shared	29.8		2.61	(-3.40, 8.63)	0.391				
Education category (Compulsory-vocational)	27.6								
• Upper secondary	29.0		1.42	(-4.62, 7.45)	0.643				
• University	31.4		3.85	(-2.42,10.12)	0.226				
Employment (unemployed)	30.6								
• Employed	29.3		-1.35	(-4.33, 1.63)	0.371				
Partnership	25.5								
• Yes	30.0		4.54	(-0.22, 9.31)	0.061	2.48	(-2.11, 7.08)	0.287	
Migration background (None)	29.7								
• With migration	31.0		1.35	(-2.40, 5.10)	0.475				
Religion (no)	30.3								
• Yes	28.3		-1.96	(-4.77, 0.85)	0.169				
Time since death									
• Continuous	-		0.01	(-0.22, 0.24)	0.909				
*Cancer-related characteristics*									
Sex of the child (male)	29.7								
• Female	29.9		0.22	(-2.40, 2.83)	0.871				
Age at death									
• Continuous	-		0.08	(-0.13, 0.30)	0.435				
Diagnosis at death (CNS tumor)	28.9								
• Leukemia/lymphoma	30.6		-1.69	(-4.89, 1.51)	0.298				
• Other solid tumors	30.2		-0.47	(-3.97, 3.04)	0.792				
Location of death (health facility)	29.2								
• Home	30.3		1.10	(-1.54,3.75)	0.411				

*Categories inside parenthesis “(category)” are the reference group. *p*-values < 0.05 are in **BOLD**

^1^Univariable linear regression using Resilience sum score as dependent (outcome) variable and sociodemographic and cancer-related characteristics as independent (predictor) variable

^2^Multivariable linear regression included independent (predictor) variables from univariable regression with *p* < 0.10

^3^Based on marginal means of univariable linear regression

We plotted a spline to determine non-linear association between time since death and resilience sum score. Overall, we found no change in resilience sum score over time since death (Appendix Figure S2).

### Association of psychological distress and prolonged grief disorder with resilience (Aim 3)

Lower resilience scores were associated with higher psychological distress (Table 3). For the global severity index (GSI) as an outcome, each unit increase in resilience was associated with a reduction in GSI by 0.71 (adjusted β = -0.71, 95% CI: -0.96, -0.46, *p* < 0.001). Similarly, depression (adjusted β = -0.74, 95% CI: -0.99, -0.49, *p* < 0.001), anxiety (adjusted β = -0.63, 95% CI: -0.88, -0.38, *p* < 0.001), and somatization (adjusted β = -0.44, 95% CI: -0.69, -0.20, *p* < 0.001) were inversely associated with resilience scores.

**Table 3:** Association of resilience (CD-RISC 10) with psychological distress (BSI-18) and prolonged grief disorder (PG-13) in bereaved parents

	Crude			Adjusted*		
Outcome	Coefficient	95% Confidence interval	*p*-value	Coefficient	95% Confidence interval	*p*-value
Global severity index^1^	-0.70	(-0.94,-0.45)	< 0.001	-0.71	(-0.96,-0.46)	< 0.001
Depression^1^	-0.68	(-0.93,-0.43)	< 0.001	-0.74	(-0.99,-0.49)	< 0.001
Anxiety^1^	-0.66	(-0.91,-0.42)	< 0.001	-0.63	(-0.88,-0.38)	< 0.001
Somatization^1^	-0.45	(-0.68,-0.21)	< 0.001	-0.44	(-0.69,-0.20)	< 0.001
**Outcome**	**Odds ratio**	**95% Confidence interval**	***p*** **-value**	**Odds ratio**	**95% Confidence interval**	***p*** **-value**
Psychological distress (case)^2^	0.83	(0.75, 0.93)	0.001	0.83	(0.74, 0.93)	0.002
Prolonged grief disorder	0.76	(0.63, 0.91)	0.003	0.73	(0.58, 0.92)	0.008

*Adjusted for age, sex, and time since death

^1^Using linear regression, psychological distress was fitted with T scores of global severity index, somatization, anxiety, and depression as continuous dependent variables, and resilience sum score as continuous independent variable

^2^ Using logistic regression, caseness (BSI-18) and prolonged grief disorder were modeled as the dichotomous dependent variable, while the resilience sum score was treated as a continuous independent variable

Higher resilience was associated with a lower likelihood of psychological distress (adjusted OR = 0.83, 95% CI: 0.74, 0.93, *p* = 0.002) or prolonged grief disorder (adjusted OR = 0.73, 95% CI: 0.58, 0.92, *p* = 0.008) for each one-point increase in the resilience sum score (Table 3).

A summary of psychological distress (BSI-18) scores and prolonged grief disorder (PG-13) of bereaved parents can be found in Appendix Table S5.

## Discussion

Our study revealed that bereaved parents report higher resilience than CCS parents. Risk of poverty is associated with lower resilience in bereaved parents, suggesting the impact of financial vulnerabilities on resilience. Accordingly, higher resilience is associated with lower psychological distress and lower risk of prolonged grief disorder. Thus, higher resilience could be a protective factor for adverse mental health outcomes.

Bereaved parents experience intense emotions after the death of their child. The irreversibility of their loss may lead them to develop unique coping mechanisms and psychological resources to refocus their lives, and experience posttraumatic growth (“positive changes”) [29]. These positive changes can include appreciation of life, strengthening established relationships, looking for opportunities that were previously not or rarely considered, or refocusing one’s life, all of which might be associated with their resilience [29]. Resilience of CCS parents may develop differently. Coping may focus on caring for their child who had cancer and include strategies such as planning and active problem-solving, self-control, and accepting responsibility [30]. Families might face new challenges after treatment, including greater need for information on late effects and tumor recurrence, financial burden from ongoing healthcare, difficulties with school and social reintegration, and decreasing psychosocial [31]. These challenges can result in lower sense of competence (or self-efficacy) and perception of control over life events, both of which are important components of resilience [32, 33].

Several resilience-building resources, such as strong partnership and social support, were identified in the literature as contributing to resilience [2, 34]. In our study, we identified the economic situation as a major factor influencing the resilience of bereaved parents. Research shows that financial stability plays a critical role in mitigating stressors and uncertainties, particularly during the grieving process as it promotes autonomy and perception of control [35]. Access to a sufficient and stable income and savings enhances one’s ability to meet basic needs but also allows families to rebuild a sense of life normality and focus on healing [36]. For example, it may allow parents to take time off work for grieving, seek therapy, or participate in support groups [35, 36]. It allows them to have a sense of control and autonomy that is crucial for maintaining psychological wellbeing [11].

Resilience, as a construct, remained stable over time since the child’s death. Although our data were not longitudinal, this aligns with prior research suggesting that resilience is a relatively stable trait [37], though it can be modified following significant adversities [38, 39]. In comparison with CCS parents who had lower resilience, we hypothesize that the experience of losing a child may foster the development of resilience, which is subsequently maintained over time.

Spirituality is an important component of resilience [11, 40], yet the data on bereaved parents’ religiosity and spirituality is challenging to interpret. The bimodal distribution revealed that bereaved parents either believe or not in an omniscient being with clearer distinction, as compared to more mixed responses from the CCS parents. This can be interpreted that the experience of loss has a significant impact that made their beliefs clearer and delineated compared to the CCS parents. Moreover, bereaved parents reported a lower sense of purpose in life but expressed greater trust and a stronger sense of security in their relationships. This provides a more nuanced understanding of the high resilience of bereaved parents that comes from resistance (or handling negative emotions, trusting one’s instincts, and perceived benefits of stress) and positivity (having a positive attitude to change and secure relationships) that is different from CCS parents.

In line with previous research, our study revealed a strong association of resilience and mental health outcomes. Resilience has been identified as a protective factor that prevents the person from developing adverse mental health outcomes [2, 12, 13]. Bereaved parents with higher resilience tend to report better adjustment to grief and healthier psychological functioning [2, 12]. In contrast, bereaved parents with lower resilience are at increased risk of prolonged grief disorder, anxiety, and depression [12]. Factors such as age and socioeconomic status may also influence the relationship between resilience and psychological outcomes in bereaved parents [41].

### Strengths and limitations

Our study provides insights into the resilience of bereaved parents, contributing to the limited quantitative studies on this topic. Our analysis quantifies the strength and magnitude of associations and provides empirical and data-driven understanding of resilience in this population. Our registry- and population-based control group (CCS parents) was a methodological improvement from previous studies resulting in higher reliability and accuracy. To reduce potential confounding, our results were adjusted for sociodemographic differences between groups (CCS parents and bereaved parents) and restricted the analysis to the German-speaking Swiss population. This approach minimized cultural and regional influences, strengthening the internal validity of the study.

This study had several limitations that should be acknowledged in the interpretation of our findings. First, the cross-sectional nature of the analysis precluded the establishment of temporality. As such, we could not determine whether low resilience leads to prolonged grief disorder or vice versa, limiting causal interpretations. Second, our recruitment strategy does not allow us to conclude anything about recruitment rate, recruitment pattern, and selection bias. We have no information about the sociodemographic characteristics of those who were not approached by the survey. Participation could have been potentially skewed towards those with better mental health outcomes and, thus, may have resulted in an overestimation of resilience in this population. Fourth, a period bias may have affected the results due to the significant time lapse between data collection for CCS-parents and bereaved parents. The bereaved parents’ data were collected during or after the COVID-19 pandemic, while CCS-parents’ data were collected before the pandemic. This temporal discrepancy introduces a potential historical bias. Another limitation is the use of the PG-13 questionnaire prior to its 2021 revision based on DSM-V-TR criteria for prolonged grief disorder [42]. Although this may affect comparability with more recent studies, the main changes on questionnaire related to presence of death and time since loss were all implied on our respondents as these were part of the enrollment criteria [43]. As such, the use of the newer questionnaire is unlikely to have influenced the results. Moreover, Finally, while restricting the analysis to German-speaking participants reduced the confounding effect of culture, this restriction also resulted to limited generalizability to other regions in Switzerland.

### Implications of the study and outlook

Our findings emphasize the importance of understanding unique adaptive mechanisms following bereavement, as they may serve as valuable psychological resources for coping with future stress and challenges. Resilience building strategies, including cognitive behavioral therapy, mindfulness and social support interventions, could benefit both bereaved parents and parents of survivors. Our results suggest that by enhancing resilience, there could be a reduction of grief-related psychological disorders (for bereaved parents), reduction of overall distress, and thereby improve general wellbeing.

Moreover, the significant impact of financial vulnerabilities on resilience underlines the importance of addressing socio-economic determinants in bereavement care [35, 36]. Providing direct and indirect financial support to bereaved parents may enhance resilience and mitigate psychological distress and prolonged grief disorder [35, 36]. Direct financial support could include assistance over housing and daily living expenses, while indirect financial support could include subsidized counselling services or providing job security both of which could significantly benefit the bereaved parents [36]. Healthcare systems could also be improved through the establishment of comprehensive bereavement services including resilience-based screening, tailored support, and sustained connection with social workers.

## Conclusion

Bereaved parents had higher overall resilience compared to CCS parents, and risk of poverty is associated with lower resilience. Additionally, higher resilience is associated with reduced psychological distress and a lower risk of prolonged grief disorder, suggesting its protective role against adverse mental health outcomes.

## Electronic supplementary material

Below is the link to the electronic supplementary material.

Supplementary Material 1: Appendix

## Data Availability

Data can be made available upon reasonable request from the corresponding author.

## Abbreviations

BSI-18: Brief Symptom Index (18 items)
CCS: Childhood cancer survivor
CD-RISC 10: Connor-Davidson Resilience Scale (10 items)
ChCR: Swiss Childhood Cancer Registry
DSM-V-TR: Diagnostic and Statistical Manual of Mental Disorders, 5th edition, Text revision
EKNZ: Ethikkomission Nordwest- und Zentralschweiz (Ethics Commission of Northwest and Central: Switzerland)
GSI: Global severity index
ICCC-3: International Classification of Childhood Cancer, Third Edition
OR: Odds ratio
PG-13: Prolonged Grief Questionnaire (13 items)
PGD: Prolonged grief disorder
SCCSS-Parents: Swiss Childhood Cancer Survivor Study – Parents
SD: Standard deviation
STROBE: Strengthening the Reporting of Observational Studies in Epidemiology (STROBE) guidelines
