# Supplementary material for: Resilience among parents whose child died of cancer – investigating its role on psychological distress and prolonged grief disorder: results from a cross-sectional survey in Switzerland
Source: BMC Palliat Care. 2025 Aug 4;24:218. doi: 10.1186/s12904-025-01854-8 (PMC12323101; doi:10.1186/s12904-025-01854-8)
Supplement: Supplementary file 1 — Supplementary Material 1: Appendix [file 12904_2025_1854_MOESM1_ESM.docx]

**Appendix/ Online Supplement**

**Resilience among parents whose child died of cancer – Investigating its role on psychological distress and prolonged grief: Results from a cross-sectional survey in Switzerland**

Short title: Resilience of cancer bereaved parents

Peter Francis Raguindin 1, Eva De Clercq 1, Anna Katharina Vokinger 1, Eddy Carolina Pedraza 1, Céline Bolliger 1, Katrin Scheinemann 1,2, Eva Maria Tinner 3, Eva Bergstraesser 4, Andre Oscar von Bueren 5, Gisela Michel 1

1 Faculty of Health Sciences and Medicine, University of Lucerne, Lucerne, Switzerland

2 Division of Hematology-Oncology, Children’s Hospital of Eastern Switzerland, St Gallen, Switzerland

3 Division of Hematology/Oncology, Department of Pediatrics, Inselspital Bern, University of Bern, Bern, Switzerland

4 University Children’s Hospital Zurich, Zurich, Switzerland

5 CANSEARCH Research Platform for Pediatric Oncology and Hematology, Faculty of Medicine, Department of Pediatrics, Gynecology and Obstetrics, University of Geneva, Geneva, Switzerland

Corresponding author

Prof. Dr. Gisela Michel

Faculty of Health Sciences and Medicine

University of Lucerne

Alpenquai 4, 6005 Lucerne

[Gisela.Michel@unilu.ch](mailto:Gisela.Michel@unilu.ch)

STROBE Statement—checklist of items that should be included in reports of observational studies

|  | Item No | Recommendation | Page/ Line no. |
| --- | --- | --- | --- |
| **Title and abstract** | 1 | (*a*) Indicate the study’s design with a commonly used term in the title or the abstract | P1 L1  P2 L37 |
|  |  | (*b*) Provide in the abstract an informative and balanced summary of what was done and what was found | P2-3 L25-57 |
| Introduction | | |  |
| Background/rationale | 2 | Explain the scientific background and rationale for the investigation being reported | P4 L69-88 |
| Objectives | 3 | State specific objectives, including any prespecified hypotheses | P5 L90-94 |
| Methods | | |  |
| Study design | 4 | Present key elements of study design early in the paper | P5 97-99 |
| Setting | 5 | Describe the setting, locations, and relevant dates, including periods of recruitment, exposure, follow-up, and data collection | P5-6 L102-134 |
| Participants | 6 | (*a*) Give the eligibility criteria, and the sources and methods of selection of participants | P5 L102-105  P6 L122-128 |
| Variables | 7 | Clearly define all outcomes, exposures, predictors, potential confounders, and effect modifiers. Give diagnostic criteria, if applicable | P6-8 L136-184 |
| Data sources/ measurement | 8* | For each variable of interest, give sources of data and details of methods of assessment (measurement). Describe comparability of assessment methods if there is more than one group | P6-8 L136-184 |
| Bias | 9 | Describe any efforts to address potential sources of bias | P8-9 L193-196  P9 L200-201  P9 L213-214 |
| Study size | 10 | Explain how the study size was arrived at | n.a. |
| Quantitative variables | 11 | Explain how quantitative variables were handled in the analyses. If applicable, describe which groupings were chosen and why | P8-10 L186-224 |
| Statistical methods | 12 | (*a*) Describe all statistical methods, including those used to control for confounding | P8-10 L186-224 |
|  |  | (*b*) Describe any methods used to examine subgroups and interactions | n.a. |
|  |  | (*c*) Explain how missing data were addressed | L137-138  L146-148  L160 |
|  |  | (*d*) If applicable, describe analytical methods taking account of sampling strategy | n.a. |
|  |  | (*e*) Describe any sensitivity analyses | P9 L197-201 |
| Results | | |  |
| Participants | 13* | (a) Report numbers of individuals at each stage of study—eg numbers potentially eligible, examined for eligibility, confirmed eligible, included in the study, completing follow-up, and analysed | P9 L213  Appendix Table S1  Figure 1 |
|  |  | (b) Give reasons for non-participation at each stage | n.a. |
|  |  | (c) Consider use of a flow diagram | Figure 1 |
| Descriptive data | 14* | (a) Give characteristics of study participants (eg demographic, clinical, social) and information on exposures and potential confounders | Table 1 |
|  |  | (b) Indicate number of participants with missing data for each variable of interest |  |
| Outcome data | 15* | Report numbers of outcome events or summary measures | Table 2 |
| Main results | 16 | (*a*) Give unadjusted estimates and, if applicable, confounder-adjusted estimates and their precision (eg, 95% confidence interval). Make clear which confounders were adjusted for and why they were included | Table 2-4  Figure 2 |
|  |  | (*b*) Report category boundaries when continuous variables were categorized | n.a. |
|  |  | (*c*) If relevant, consider translating estimates of relative risk into absolute risk for a meaningful time period | n.a. |
| Other analyses | 17 | Report other analyses done—eg analyses of subgroups and interactions, and sensitivity analyses | Appendix Table S1-5  Appendix Figure S2 |
| Discussion | | |  |
| Key results | 18 | Summarise key results with reference to study objectives | P12 L293-297 |
| Limitations | 19 | Discuss limitations of the study, taking into account sources of potential bias or imprecision. Discuss both direction and magnitude of any potential bias | P115 L358-376 |
| Interpretation | 20 | Give a cautious overall interpretation of results considering objectives, limitations, multiplicity of analyses, results from similar studies, and other relevant evidence | P15 L374-376 |
| Generalisability | 21 | Discuss the generalisability (external validity) of the study results | P14-15 L346-364 |
| Other information | | |  |
| Funding | 22 | Give the source of funding and the role of the funders for the present study and, if applicable, for the original study on which the present article is based | P18 L444-450 |

*Give information separately for exposed and unexposed groups.

**Note:** An Explanation and Elaboration article discusses each checklist item and gives methodological background and published examples of transparent reporting. The STROBE checklist is best used in conjunction with this article (freely available on the Web sites of PLoS Medicine at http://www.plosmedicine.org/, Annals of Internal Medicine at http://www.annals.org/, and Epidemiology at http://www.epidem.com/). Information on the STROBE Initiative is available at www.strobe-statement.org.


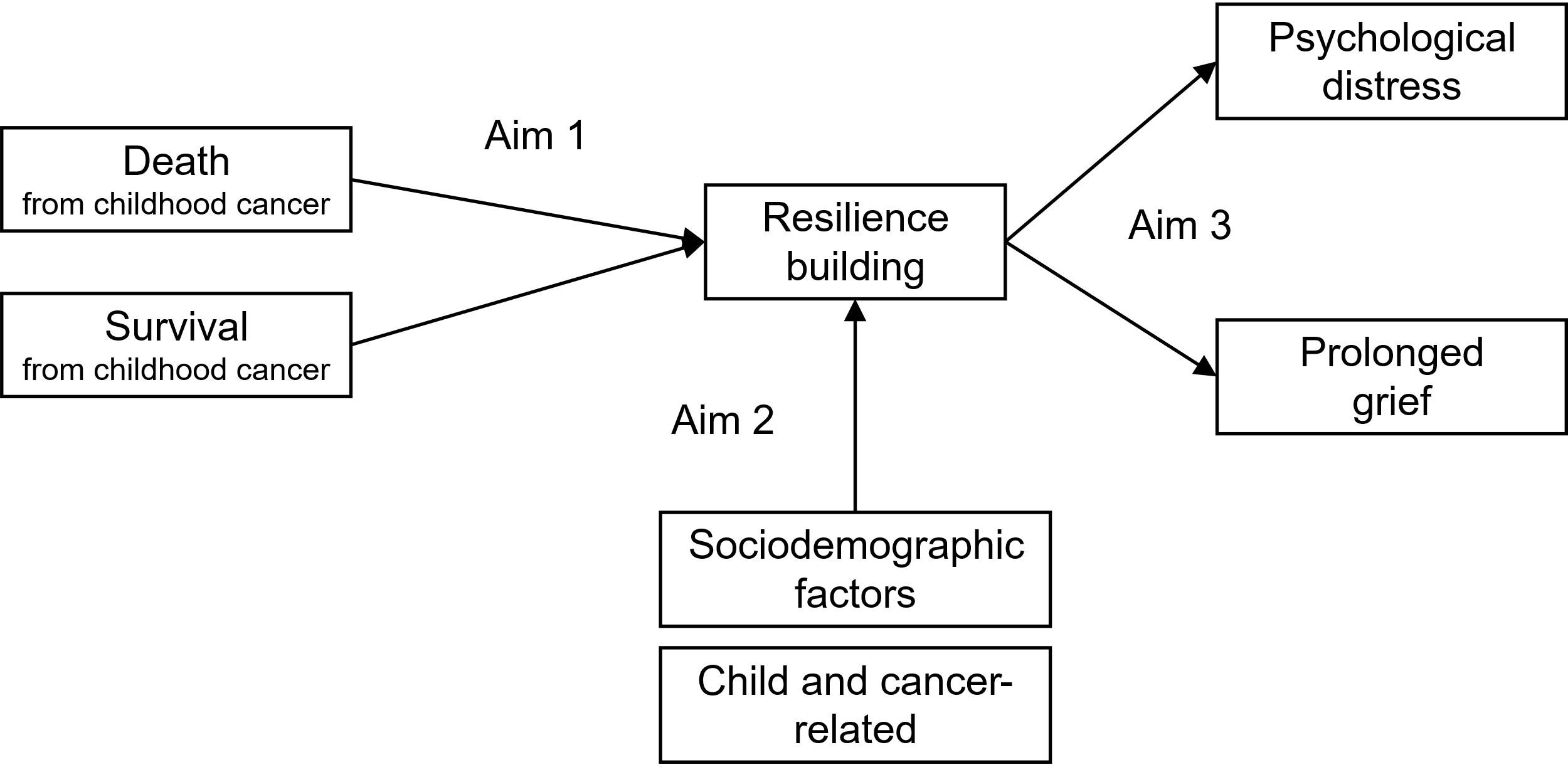


**Appendix Figure S1.** Analysis framework

**Appendix Table S1.** Item comparison to CDRISC-10 between bereaved parents and CCS parents

|  | Bereaved parents | CCS parents | P value^1^ |
| --- | --- | --- | --- |
| Number of participants (N) | 103 | 345 |  |
| I am able to adapt when changes occur. (Positivity) |  |  |  |
| Not at all | 1 (1.0%) | 3 (0.9%) | 0.011 |
| Rarely true | 4 (3.9%) | 4 (1.2%) |  |
| Sometime true | 12 (11.7%) | 58 (16.8%) |  |
| Often true | 29 (28.2%) | 144 (41.7%) |  |
| Always true | 57 (55.3%) | 136 (39.4%) |  |
| Item mean, mean (SD)^2^ | 3.3 (0.9) | 3.2 (0.8) | 0.102 |
| I can deal with whatever comes my way. (Positivity) |  |  |  |
| Not at all | 3 (2.9%) | 10 (2.9%) | <0.001 |
| Rarely true | 5 (4.9%) | 18 (5.2%) |  |
| Sometime true | 18 (17.5%) | 101 (29.3%) |  |
| Often true | 41 (39.8%) | 174 (50.4%) |  |
| Always true | 36 (35.0%) | 42 (12.2%) |  |
| Item mean, mean (SD)^2^ | 3.0 (1.0) | 2.6 (0.9) | <0.001 |
| I try to see the humorous side of things when I am faced with problems. (Resistance) |  |  |  |
| Not at all | 3 (2.9%) | 19 (5.5%) | 0.032 |
| Rarely true | 9 (8.7%) | 54 (15.7%) |  |
| Sometime true | 41 (39.8%) | 129 (37.4%) |  |
| Often true | 28 (27.2%) | 105 (30.4%) |  |
| Always true | 22 (21.4%) | 38 (11.0%) |  |
| Item mean, mean (SD)^2^ | 2.6 (1.0) | 2.3 (1.0) | 0.011 |
| Having to cope with stress can make me stronger. (Resistance) |  |  |  |
| Not at all | 6 (5.8%) | 17 (4.9%) | 0.213 |
| Rarely true | 12 (11.7%) | 50 (14.5%) |  |
| Sometime true | 33 (32.0%) | 133 (38.6%) |  |
| Often true | 37 (35.9%) | 119 (34.5%) |  |
| Always true | 15 (14.6%) | 26 (7.5%) |  |
| Item mean, mean (SD)^2^ | 2.4 (1.1) | 2.3 (1.0) | 0.136 |
| I tend to bounce back after illness, injury or other hardships. (Positivity) |  |  |  |
| Not at all | 0 (0.0%) | 1 (0.3%) | 0.123 |
| Rarely true | 3 (2.9%) | 5 (1.4%) |  |
| Sometime true | 15 (14.6%) | 24 (7.0%) |  |
| Often true | 41 (39.8%) | 148 (42.9%) |  |
| Always true | 44 (42.7%) | 167 (48.4%) |  |
| Item mean, mean (SD)^2^ | 3.2 (0.8) | 3.4 (0.7) | 0.061 |
| I believe I can achieve my goals, even if there are obstacles. (Competence) |  |  |  |
| Not at all | 0 (0.0%) | 2 (0.6%) | 0.181 |
| Rarely true | 1 (1.0%) | 7 (2.0%) |  |
| Sometime true | 15 (14.6%) | 75 (21.7%) |  |
| Often true | 50 (48.5%) | 173 (50.1%) |  |
| Always true | 37 (35.9%) | 88 (25.5%) |  |
| Item mean, mean (SD)^2^ | 3.2 (0.7) | 3.0 (0.8) | 0.013 |
| Under pressure, I stay focused and think clearly. (Resistance) |  |  |  |
| Rarely true | 3 (2.9%) | 12 (3.5%) | 0.019 |
| Sometime true | 21 (20.4%) | 108 (31.3%) |  |
| Often true | 40 (38.8%) | 145 (42.0%) |  |
| Always true | 39 (37.9%) | 80 (23.2%) |  |
| Item mean, mean (SD)^2^ | 3.1 (0.8) | 2.8 (0.8) | 0.004 |
| I am not easily discouraged by failure. (Competence) |  |  |  |
| Not at all | 2 (1.9%) | 8 (2.3%) | 0.415 |
| Rarely true | 8 (7.8%) | 26 (7.5%) |  |
| Sometime true | 23 (22.3%) | 103 (29.9%) |  |
| Often true | 47 (45.6%) | 155 (44.9%) |  |
| Always true | 23 (22.3%) | 53 (15.4%) |  |
| Item mean, mean (SD)^2^ | 2.8 (0.9) | 2.6 (0.9) | 0.143 |
| I think of myself as a strong person when dealing with life’s challenges and difficulties. (Competence) |  |  |  |
| Not at all | 1 (1.0%) | 3 (0.9%) | 0.110 |
| Rarely true | 3 (2.9%) | 13 (3.8%) |  |
| Sometime true | 15 (14.6%) | 83 (24.1%) |  |
| Often true | 45 (43.7%) | 157 (45.5%) |  |
| Always true | 39 (37.9%) | 89 (25.8%) |  |
| Item mean, mean (SD)^2^ | 3.1 (0.8) | 2.9 (0.9) | 0.016 |
| I am able to handle unpleasant or painful feelings like sadness, fear, and anger. (Resistance) |  |  |  |
| Not at all | 2 (1.9%) | 2 (0.6%) | 0.079 |
| Rarely true | 1 (1.0%) | 17 (4.9%) |  |
| Sometime true | 23 (22.3%) | 86 (24.9%) |  |
| Often true | 44 (42.7%) | 163 (47.2%) |  |
| Always true | 33 (32.0%) | 77 (22.3%) |  |
| Item mean, mean (SD)^2^ | 3.0 (0.9) | 2.9 (0.8) | 0.090 |

^1^p value from chi-squared test, unless specified otherwise. CCS, childhood cancer survivor; SD, standard deviation

^2^ p value from students t-test, comparing means of two groups (bereaved parents and CCS parents)

**Appendix Table S2.** Comparison of clustered and non-clustered fully adjusted model comparing the resilience sum score between bereaved parents and CCS parents

|  | Non clustered model | | | Clustered model | | |
| --- | --- | --- | --- | --- | --- | --- |
|  | Coefficient | 95% CI | P value | Coefficient | 95% CI | P value |
| Population (ref-CCS Parents)  Bereaved parents | 1.77 | 0.13, 3.40 | 0.033 | 1.78 | 0.15, 3.40 | 0.032 |
| Age (in years) | 0.01 | -0.09, 0.11 | 0.852 | 0.01 | -0.09, 0.11 | 0.839 |
| Risk of poverty (ref-no risk)  At risk | -1.59 | -2.90, -0.28 | 0.017 | -1.59 | -2.89, -0.29 | 0.016 |
| Religion (ref-with)  Without religion | 0.85 | -0.71, 2.40 | 0.284 | 0.85 | -0.69, 2.39 | 0.281 |
| Migration background (ref-None)  With | 0.11 | -1.76, 1.98 | 0.905 | 0.10 | -1.75, 1.96 | 0.912 |
| Time since diagnosis (in years) | -0.001 | -0.10, 0.09 | 0.928 | -0.01 | -0.10, 0.09 | 0.912 |
| LR test with linear |  |  |  |  |  | 0.356 |

*Note that LR test comparing to linear model showed p value of 0.356 suggesting that clustering does not exist.

**Appendix Table S3.** Summary of items on religiosity and spirituality differences between parents of childhood cancer survivors and bereaved parents.

|  | CCS parents  N= 345 | Bereaved parents  N=103 | P value^1^ |  |
| --- | --- | --- | --- | --- |
| I have a strong sense of purpose in life. | | | | |
| Not at all | 7 (2.0%) | 16 (15.5%) | <0.001 |  |
| Rarely true | 23 (6.7%) | 12 (11.7%) |  |  |
| Sometime true | 61 (17.7%) | 23 (22.3%) |  |  |
| Often true | 169 (49.0%) | 24 (23.3%) |  |  |
| Always true | 85 (24.6%) | 28 (27.2%) |  |  |
| Item mean, mean (SD) | 2.9 (0.9) | 2.3 (1.4) | <0.001 |  |
| I have one close and secure relationship. | | | | |
| Not at all | 8 (2.3%) | 2 (1.9%) | 0.034 |  |
| Rarely true | 10 (2.9%) | 2 (1.9%) |  |  |
| Sometime true | 42 (12.2%) | 6 (5.8%) |  |  |
| Often true | 94 (27.2%) | 18 (17.5%) |  |  |
| Always true | 191 (55.4%) | 75 (72.8%) |  |  |
| Item mean, mean (SD) | 3.3 (0.9) | 3.6 (0.8) | 0.010 |  |
| Sometimes fate or God helps me | | | | |
| Not at all | 103 (29.9%) | 44 (42.7%) | 0.017 |  |
| Rarely true | 70 (20.3%) | 15 (14.6%) |  |  |
| Sometime true | 61 (17.7%) | 12 (11.7%) |  |  |
| Often true | 63 (18.3%) | 11 (10.7%) |  |  |
| Always true | 48 (13.9%) | 21 (20.4%) |  |  |
| Item mean, mean (SD) | 1.7 (1.4) | 1.5 (1.4) | 0.374 |  |

^1^p value from chi squared test

Abbreviation: CCS, childhood cancer survivor; SD. standard deviation

**Appendix Table S4.** Univariable clustered regression on the association of Resilience sum score and sociodemographic-, child- and cancer-related characteristics

|  | Coefficient | 95% confidence interval | P value |
| --- | --- | --- | --- |
| Sociodemographic characteristics | | | |
| Sex (male) |  |  |  |
| - Female | -1.28 | (-3.69, 1.13) | 0.297 |
| Age (<50 years) |  |  |  |
| - 50-60 years | 1.44 | (-1.61, 4.50) | 0.355 |
| - >60 years | 2.25 | (-1.27, 5.76) | 0.210 |
| Risk of poverty (low risk) |  |  |  |
| - High-risk | -8.17 | (-11.84,-4.50) | <0.001 |
| Living arrangement |  |  |  |
| - Shared | 2.52 | (-3.40, 8.45) | 0.404 |
| Education category (Compulsory-vocational) |  |  |  |
| - Upper secondary | 1.41 | (-4.35, 7.17) | 0.632 |
| - University | 4.10 | (-1.93,10.13) | 0.182 |
| Employment (unemployed) |  |  |  |
| - Employed | -1.16 | (-3.89, 1.57) | 0.404 |
| Partnership |  |  |  |
| - Yes | 4.48 | (-0.21, 9.18) | 0.061 |
| Migration background (None) |  |  |  |
| - With migration | 1.22 | (-2.41, 4.85) | 0.510 |
| Religion (no) |  |  |  |
| - Yes | -1.95 | (-4.75, 0.85) | 0.172 |
| Time since death |  |  |  |
| - Continuous | 0.02 | (-0.23, 0.27) | 0.871 |
| Child- and cancer-related characteristics | | | |
| Sex of the child (male) |  |  |  |
| - Female | 0.32 | (-2.42, 3.06) | 0.819 |
| Age at death |  |  |  |
| - Continuous | 0.10 | (-0.13, 0.33) | 0.405 |
| Diagnosis at death (CNS tumor) |  |  |  |
| - Non-CNS tumor | -1.63 | (-4.93, 1.66) | 0.331 |
| - others | -0.58 | (-4.21, 3.04) | 0.752 |
| Location of death (health facility) |  |  |  |
| - Home | 1.03 | (-1.69, 3.75) | 0.457 |

**Appendix Table S5.** Summary of psychological distress and prolonged grief in the study population

|  | Bereaved parents |
| --- | --- |
|  | (N=103) |
| Psychological distress (BSI-18) | |
| Global Severity Index | 50.3 (9.4) |
| Somatization | 49.7 (8.5) |
| Anxiety | 47.6 (9.4) |
| Depression | 52.1 (9.5) |
| Caseness |  |
| No | 92 (89.3%) |
| Yes | 11 (10.7%) |
| Prolonged grief disorder (PG-13) | |
| Prolonged grief disorder |  |
| No | 98 (95.1%) |
| Yes | 5 (4.9%) |
| Separation distress |  |
| No | 79 (76.7%) |
| Yes | 24 (23.3%) |
| Duration criterion |  |
| No | 63 (61.2%) |
| Yes | 40 (38.8%) |
| Symptom criterion |  |
| No | 103 (94.2%) |
| Yes | 6 (5.8%) |
| Impairment criteria |  |
| No | 88 (85.4%) |
| Yes | 15 (14.6%) |

20

25

30

35

40

Resilience sum score

0

5

10

15

20

25

Time since bereaved (years)

**Appendix Figure S2.** Spline model of resilience scores as a function of time since death for bereaved parents
